# Lack of Impacts during Early Establishment Highlights a Short-Term Management Window for Minimizing Invasions from Perennial Biomass Crops

**DOI:** 10.3389/fpls.2017.00767

**Published:** 2017-05-15

**Authors:** Natalie M. West, David P. Matlaga, Ranjan Muthukrishnan, Greg Spyreas, Nicholas R. Jordan, James D. Forester, Adam S. Davis

**Affiliations:** ^1^Pest Management Research Unit, United States Department of Agriculture – Agricultural Research Service, SidneyMT, USA; ^2^Global Change and Photosynthesis Research Unit, United States Department of Agriculture – Agricultural Research Service, UrbanaIL, USA; ^3^Department of Biology, Susquehanna University, SelinsgrovePA, USA; ^4^Department of Fisheries, Wildlife, and Conservation Biology, University of Minnesota, St. PaulMN, USA; ^5^Prairie Research Institute, Illinois Natural History Survey, ChampaignIL, USA; ^6^Department of Agronomy and Plant Genetics, University of Minnesota, St. PaulMN, USA

**Keywords:** biomass crops, *Miscanthus*, impacts, controlled invasions, agroecosystems

## Abstract

Managing intentional species introductions requires evaluating potential ecological risks. However, it is difficult to weigh costs and benefits when data about interactions between novel species and the communities they are introduced to are scarce. In anticipation of expanded cultivation of perennial biomass crops, we experimentally introduced *Miscanthus sinensis* and *Miscanthus* × *giganteus* (two non-native candidate biomass crops) into two different non-crop habitats (old field and flood-plain forest) to evaluate their establishment success and impact on ambient local communities. We followed these controlled introductions and the composition dynamics of the receiving communities over a 5-year period. Habitats differed widely in adult *Miscanthus* survival and reproduction potential between species, although seed persistence and seedling emergence were similar in the two biomass crops in both habitats. Few introductions survived in the floodplain forest habitat, and this mortality precluded analyses of their potential impacts there. In old field habitats, proportional survival ranged from 0.3 to 0.4, and plant survival and growth increased with age. However, there was no evidence of biomass crop species effects on community richness or evenness or strong impacts on the resident old field constituents across 5 years. These results suggest that *Miscanthus* species could establish outside of cultivated fields, but there will likely be a lag in any impacts on the receiving communities. Local North American invasions by *M. sinensis* and *M. sacchariflorus* display the potential for *Miscanthus* species to develop aggressively expanding populations. However, the weak short-term community-level impacts demonstrated in the current study indicate a clear management window in which eradicating species footholds is easily achieved, if they can be detected early enough. Diligent long-term monitoring, detection, and eradication plans are needed to successfully minimize harmful invasions from these biomass crops.

## Introduction

Evaluating ecological risks associated with intentional plant introductions requires understanding species colonization and establishment success as well as potentially negative impacts on the recipient community (Theoharides and Dukes, 2007; Barney et al., 2013). For agricultural introductions, the disadvantages of a high escape probability may be mitigated by low potential impacts of that species on surrounding communities (Yokomizo et al., 2012; Grechi et al., 2014). However, stochasticity, agricultural breeding, and novel selection pressures in response to new interactions and environments can complicate predictions about the relative benefits versus threat potential of novel introductions (Mack, 2000; Moles et al., 2012; Richardson, 2013; Driscoll et al., 2014). Therefore, estimated responses may not be directly comparable across systems. Species traits and invasion history can provide a preliminary indication of how an introduction will fare (Davis et al., 2010), but species success and consequences depend on the spatial and temporal context and interactions with the receiving community (Hulme et al., 2013; Kumschick et al., 2015). To better predict potential costs associated with the cultivation and introduction of novel species, we need *in situ* empirical information on the establishment likelihood and community impacts associated with introductions (Flory et al., 2012; Scasta et al., 2015).

We measured the establishment success and impact (measureable change in ecological properties or processes, i.e., Simberloff et al., 2013; Blackburn et al., 2014) of *Miscanthus* introductions in two common non-agronomic habitats in central Illinois, USA. *Miscanthus* sp. have been widely introduced for horticulture, and more recently as biomass feedstocks, in the US and Europe since at least the 19th century. Escaped patches already present in the landscape tend to be small (<1 m^2^), but there are locally extensive populations (Quinn et al., 2010; Dougherty et al., 2014; Schnitzler and Essl, 2015). We chose old field and floodplain forest habitats for two main reasons. First, these are dominant non-agricultural plant habitats in central Illinois. Forested floodplain areas have remained largely uncultivated, whereas old field sites are often located on farmland too unproductive to remain in cultivation. Second, both habitat types are commonly found adjacent to production areas, and for this reason are likely to be receptor habitats for *Miscanthus* escaping from production fields. Quantifying the likelihood of escapes surviving and reproducing in these receptor habitats provides a context for identifying potential costs and management associated with introducing *Miscanthus* production into the central Illinois landscape.

We know that many traits attractive for biomass crops are also associated with successful invasive species (Raghu et al., 2011; Flory et al., 2012; Schnitzler and Essl, 2015). *Miscanthus* taxa display a range of characteristics associated with invaders, such as rapid biomass accumulation, tall stature, allelopathic properties, and wide ecological tolerances and dispersal capabilities (Chou, 2009; Quinn et al., 2012; Matlaga and Davis, 2013; Hager et al., 2014; Hedìnec et al., 2014), suggesting the possibility of negative impacts once plants are established. However, whether such traits will allow *Miscanthus* to establish, and significantly change the composition or functioning of receptor communities, requires evaluation (Drenovsky et al., 2012; Barney et al., 2013; Blackburn et al., 2014; Dick et al., 2014).

Impacts from non-native species are strongly context dependent and variable in magnitude and direction, which complicates assessments of new introductions (Byers and Noonburg, 2003; Pyšek et al., 2012; Hulme et al., 2013; Blackburn et al., 2014; Grechi et al., 2014). It can be difficult to distinguish invader impacts from other concurrent and potentially synergistic stressors (Dick et al., 2014; Kumschick et al., 2015). For instance, invader density likely influences variation in community interactions and impacts, but ecological impacts do not necessarily increase linearly with the density or perceived competitive dominance of the invader (Thiele et al., 2010; Jackson et al., 2015). Assessing multiple measures of introduction consequences in different environmental contexts is therefore vital for predicting the likelihood and impacts of invasion success.

We experimentally examined the establishment and impact of *Miscanthus* introduced at different densities into old fields and floodplain forest sites to: (1) quantify the viability of escapes into these habitats; (2) identify potential limitations and catalysts to *Miscanthus* establishment in non-crop habitats; and (3) evaluate the impacts of *Miscanthus* introduction on resident plant communities. We followed the long-term persistence of both clonal, seed-infertile, *Miscanthus* × *giganteus* “Illinois” clone (low risk, i.e., Quinn et al., 2015) and feral, seed-fertile, *Miscanthus sinensis* (high risk) to represent a spectrum of the potential invasiveness in *Miscanthus* germplasm being improved for biomass production. Escape viability and potential ecological limitations were evaluated by tracking recruitment, persistence, and growth of introduced plants over time. Impacts were assessed as measureable differences in species richness and evenness (components of diversity), as well as shifts in species and functional group abundance (measures of biotic interaction), between plots with and without *Miscanthus* introduced at different densities. Assessing the viability and negative impacts of non-native species introductions is important to inform prioritization and implementation of control strategies (Lewis and Porter, 2014). Our study is one of only a few to track metrics of community change over multiple years in response to controlled invasions into natural areas.

## Materials and Methods

### Study Species

*Miscanthus sinensis* Andress. is a seed-fertile crop introduced from Japan for horticultural use in the 1800s. It became naturalized in the eastern U.S. by the mid-20th century, and is locally invasive (Quinn et al., 2010). *M. sinensis* is both a candidate biomass crop as well as a parent species to other candidate varieties (e.g., Arnoult and Brancourt-Hulmel, 2015). We collected *M. sinensis* root-stock and seeds from roadside and forest opening patches in Daniel Boone National Forest, Powell County, KY, USA, in September 2009. *Miscanthus* × *giganteus* ‘Illinois’ clone (hereafter, *M. giganteus*) J.M. Greef & Deuter ex Hodkinson & Renvoize is a seed infertile hybrid of *M. sinensis* and *M. sacchariflorus* (Christian and Haase, 2001), and is one of the most widely planted cellulosic biofeedstock in the U.S. (Anderson et al., 2015). We obtained *M. giganteus* root stock for experimental plantings from the Chicago Botanic Garden. Utilizing plugs rather than seeds allowed us to evaluate plant survival and provide an estimate of long-term persistence once plants were introduced, while controlling accidental seed introductions into our study communities. We also estimated seed-based recruitment and overwintering persistence within our plots. Because the ‘Illinois’ clone is seed-infertile, we obtained seeds harvested from a pilot plantation of a pre-release, seed-fertile, tetraploid *M. giganteus* cultivar (‘PowerCane,’ Mendel Biotechnology, Hayward, CA, USA; see references in Bonin et al., 2017) to evaluate the potential for seed-based recruitment into study areas. Multiple studies have examined the seed and seedling viability and persistence of both ‘PowerCane’ and *M. sinensis* under various conditions and in various habitats (e.g., Smith and Barney, 2014; West et al., 2014a; Hager et al., 2015a; Smith et al., 2015; Bonin et al., 2017); these studies provide an understanding of the demographic contributions of seed to *Miscanthus* invasion potential. For our purposes, seed-based recruitment provides an additional measure of habitat suitability for escapes.

### Experimental Plantings

We established *Miscanthus* in three old field and three floodplain forest sites. Our old field habitats, Phillips and Trelease Prairies and the Vermillion River Observatory, and two floodplain forest sites, Nanney and Richter Tracts, are owned and managed by the University of Illinois Urbana–Champaign. The last floodplain forest habitat, Homer Lake, is part of the Champaign County, IL Park District. Consistent with management practices in our region, old field sites were mowed annually in the spring to a height of 7.5–10 cm to inhibit woody encroachment. Floodplain forests were unmanaged and subject to frequent and occasionally prolonged flooding.

Both *Miscanthus* species were propagated in the greenhouse prior to planting. We divided potted *Miscanthus* into approximately 10-cm diameter plugs with 10 to 15-cm long shoots. We hardened them off for a week and then transported them to the field. We introduced plugs into eight 10-m × 10-m single-species plots per site (four plots per species) in a split-split plot design (Supplement Figure 1A). Main single-species plots were divided into four 5-m × 5-m subplots that were each randomly assigned one of four density treatments: high (*n* = 16 plants with 1-m spacing, 1 plant/m^2^); medium (*n* = 9 plants with 1.25-m spacing, 0.56 plant/m^2^); low (*n* = 4 plants with 1.67-m spacing, 0.25 plant/m^2^); or control (*n* = 0 plants/m^2^). Plantings were positioned a minimum of 1-m from the subplot edge, and planting layouts were centered within the subplots (Supplement Figure 1B).

Introductions were initiated in April 2010. Plugs were planted into 25 cm deep holes and covered with a 25-cm × 25-cm heavy-gauge plastic mesh secured with sod staples to prevent movement due to flooding or animals. To maintain density treatments, plants that did not resprout by the spring census in 2011 and 2012 were removed and replaced. Nearly 75% of plugs (1037 out of 1392) were replanted in 2011 due to mortality; only 27% (379 out of 1392) were replanted in 2012. To control for effects of planting disturbance on community comparisons, we did sham plantings, which consisted of digging similar sized holes within the control subplots and then replacing the soil. The number of sham plantings was set as the median number of plantings (or replantings) out of all densities within that plot. We minimized soil disturbance during introduction, and any excess soil remaining after planting was transported out of the plot. Because *Miscanthus* establishment is sensitive to water limitation (Zub and Brancourt-Hulmel, 2010; Anderson et al., 2015), plugs were watered at the time of planting (or replanting), and periodically for the following month. Therefore, our data on establishment reflect a best-case scenario in terms of moisture conditions.

### Habitat Characteristics and Establishment

We measured a combination of soil fertility and soil water conditions to represent abiotic habitat differences. To quantify soil fertility, we collected soil samples to a depth of 10 cm from the center of each subplot with a 10 cm diameter soil corer in summer 2011. These samples were dried and sieved to remove non-soil particles, and analyzed by A&L Great Lakes Laboratories for plant macro- and micronutrients (P, K, Mg, Ca, S, Zn Mn, Fe, Cu, NO_3_^-^, NH_4_^+^, and B), pH, soil organic matter, and cation exchange capacity (CEC). We measured soil redox potential as an integrated measure of saturated soil water conditions over the growing season using the Indicator of Reduction in Soil (IRIS) procedure (Castenson and Rabenhorst, 2006; Jenkinson and Franzmeier, 2006). We left IRIS tubes in the control subplot of each plot from April to August 2014, and recorded the amount of ferrihydrite paint lost from the tube surface at the end of the season. To quantify surface area exposed, we took digital images of each plane of the tube surface, and combined the multiple views into a single image. We then adjusted combined images to a consistent size (2745 × 675 pixels), and counted the number of pixels that lacked paint using MATLAB (Mathworks, Natick, MA, USA). We used these counts to calculate the percent paint surface area lost to redox reactions. Additionally, we did a pulse measurement of soil water content by sampling two 10-cm soil cores per sub-subplot 24–36 h after a rain of more than 2.5 cm in July 2013. We weighed wet samples, dried them for 72 h, took the dry weight, and subtracted the difference to estimate gravimetric soil moisture. These two measurements allowed us to compare relative differences in soil water status among plots.

To simplify the inclusion of soil nutrient conditions in the examination of habitat differences, and to account for strong covariance among the soil nutrients, we created a composite soil variable. We identified the optimal group of uncorrelated soil variables necessary to adequately distinguish habitats using a linear discriminant analysis (*subselect* R package: *ldaHmat* and *eleaps* functions). Improvement in correlation with a first canonical axis peaked with four soil factors (Mg, CEC, Fe, and B), and these variables successfully predicted habitat membership with less than a 5% error. Therefore, we combined them into a composite soil fertility variable quantified as the first axis scores from a principal components analysis of the four identified variables.

To represent relative differences associated with light availability within our two habitats, we evaluated light conditions both above and below understory vegetation (vegetation cover below any existing tree canopy). We measured photosynthetically active radiation above the understory canopy (approximately 1.5 m from the ground, in μmol m^-2^ s^-1^, PARA) and light transmittance (% PARA). These factors were quantified at the subplot level, and then averaged for analysis at the plot level. We measured both above and below understory canopy PAR with a linear ceptometer (LP-80 Accu-PAR, Decagon Devices, Court Pullman, WA, USA) as the average PAR at four points around each subplot. We quantified transmittance (the amount of above understory PAR penetrating to ground level) as below understory PAR/above understory PAR.

### *Miscanthus* Recruitment

To test habitat type effects on seedling emergence, we established caged plots (to deter herbivory and seed predation) in one randomly selected corner of each plot in late fall 2011 (Supplement Figure 1B). Seeds were planted in 10 cm × 10 cm seed trays filled to a depth of 5 cm with soil from the receiving site. Each tray was placed in a cylindrical 1-mm mesh cage 40 cm in diameter and 30 cm high, which was additionally filled with site-collected soil to allow the seed tray to lie flush with the surrounding soil surface. The base of each cage was buried approximately 10 cm and secured in place. Because of site-use and material transfer agreement restrictions, we were unable to plant the seed-fertile *M. giganteus* in the field. However, previous work on the regeneration niche of this pre-release cultivar indicates fertile *M. giganteus* seed performs similarly to *M. sinensis* (Smith and Barney, 2014; West et al., 2014a). We monitored seedling emergence monthly March–November 2012, and seedlings were removed after each count to avoid confounding measures of emergence and survival.

To examine habitat type effects on seed viability after overwintering, additional seeds were cold-stratified *in situ* within stainless steel mesh packets buried in the field next to seedling plots in November 2012 (Supplement Figure 1B). Because it did not require field germination, we were able to use both species of *Miscanthus* for this test. We staked each 20 cm × 20 cm 0.5-mm wire mesh bag containing 100 seeds of the appropriate species in each corresponding subplot in November 2012. Bags were placed on bare ground, and any detritus moved to accommodate the bag was replaced to emulate site conditions. Bags remained in place until late April 2013, when they were collected and germinable seed fraction determined by counting the number of overwintered seeds that germinated under greenhouse conditions.

### *Miscanthus* Performance

We recorded plant survival and tiller number twice annually from spring 2010 to 2014 to quantify plant performance and the integrity of density treatments over time. Census timing during the year was variable due to phenological fluctuation; thus, spring measurements occurred in April–May, and fall in October–November. Per year plant measurements, such as growth and survival, involved the period from spring to spring each year. In 2014, the second census was conducted in late July to optimize eradication efforts.

In 2014, we also quantified *Miscanthus* biomass within plots. We clipped and weighed all aboveground *Miscanthus* biomass per individual plant in the field. A subset of these plants were taken back to the lab, dried for 48 h at 45 degrees Celsius, and weighed to determine the relationship between field and dry weights (see footnote to **Table 1**). Additionally, we measured the area covered by each plant by measuring the widest axis of tiller extent and the one perpendicular to it, and calculating the area as an ellipse. Any flowering panicles produced in 2013 were collected, and the number of caryopses produced by habitat and by plant were quantified by weight relative to a 100 caryopsis weight standard. Viable seed production by *M. giganteus* ‘Illinois’ clone is inhibited by incomplete gametophyte development that results in sterility (Słomka et al., 2012). Therefore, these estimates merely represent a quantification of potential seed production within these habitats given the possibility of fertile genotypes being introduced for agronomic purposes (i.e., Bonin et al., 2017). Because our management agreements required panicle collection before dehiscence (to avoid unintentional spread and naturalization at the study sites), we were unable to reliably quantify viable seed produced for *M. sinensis*, and can only present relative differences in reproductive effort.

**Table 1:** *Miscanthus* recruitment and establishment: Mean ± SD, as well as the range (in parentheses, minimum–maximum) of measurements in old field and floodplain forest plots.

	*M. giganteus*		*M. sinensis*	
		
	Old field	Floodplain	Old field	Floodplain
**(A) Recruitment**				
(Proportion seeds to seedlings)				
Overwintering	0.23 ± 0.08^1^	0.23 ± 0.09^1^	0.32 ± 0.08	0.36 ± 0.1
	(0.03 - 0.34)	(0.08 - 0.39)	(0.18 - 0.41)	(0.20 - 0.49)
Field emergence	^∗^	^∗^	0.12 ± 0.07	0.26 ± 0.15
	^∗^	^∗^	(0 - 0.28)	(0 - 0.56)
**(B) Establishment**				
*Per plot*				
Survival	0.29 ± 0.13	0.04 ± 0.05	0.38 ± 0.17	0.02 ± 0.04
	(0.11 - 0.48)	(0 - 0.18)	(0.05 - 0.65)	(0 - 0.11)
# Surviving plants^2,3^	17.8 ± 6.8	3.9 ± 2.3	19.3 ± 7.2	4 ± 2.6
	(8 - 27)	(2 - 8)	(3 - 27)	(1 - 6)
Biomass (g)^4,5^	326.8 ± 288.4	24.8 ± 21.4	168.5 ± 131.8	5.7 ± 5.6
	(1.51 - 3540)	(0.20 - 81.0)	(1 - 2160)	(0.32 - 27.1)
Area occupied by *Miscanthus* (m^2^)	7.2 ± 4.6	1.0 ± 0.7	5.3 ± 4.1	0.53 ± 1.0
	(2.4 - 18.2)	(0 - 2.0)	(0.9 - 13.8)	(0 - 2.4)
# Flowering plants	5.1 ± 2.8	^∗^	12.3 ± 6.4	^∗^
	(1 - 10)		(1 - 25)	
**Per plant**				
Area (m^2^)	0.33 ± 0.2	0.37 ± 0.2	0.24 ± 0.2	0.35 ± 0.2
	(<0.01 - 1.61)	(0.02 - 0.91)	(<0.01 - 1.2)	(0.04 - 1.3)
# Tillers	7.8 ± 4.5	2.6 ± 1.3	12.4 ± 6.8	3.3 ± 1.9
	(1 - 47)	(1 - 6)	(1 - 96)	(1 - 9)
# Inflorescences	2.2 ± 0.7	^∗^	5.2 ± 3.0	^∗^
	(1 - 8)		(1 - 37)	
# Caryopses	5552.9 ± 3605.1	^∗^	7890.9 ± 6864.8	^∗^
	(482 - 37949)		(67 - 108767)	


*^∗^Indicates metrics for which data are unavailable. Recruitment was measured over a 1 year period between 2012 and 2013; we quantified Establishment in 2014. ^1^Estimates reported for seed-fertile *M. giganteus* ‘PowerCane,’ as the ‘Illinois’ clone is seed-infertile. ^2^# Plots with surviving plants: *M. giganteus*: *n* = 12 (field) and 7 (floodplain); *M. sinensis*: *n* = 12 (field) and 3 (Floodplain). ^3^# Live plants total: *M. giganteus*: *n* = 213 (field) and 27 (floodplain); *M. sinensis*: *n* = 230 (field) and 12 (floodplain). ^4^*M. giganteus* dry weight (*g*) = 0.29^∗^field weight + 0.39; *p* < 0.001; *r*^*2*^ = 0.98. ^5^*M. sinensis* dry weight (*g*) = 0.31^∗^field weight – 0.30; *p* < 0.001; *r*^*2*^ = 0.99.*

### *Miscanthus* Impacts on the Local Plant Community

Plant community data were obtained by randomly sampling a total of 2 m^2^ within each density subplot in late June-early July 2011–2014. Each year, we quantified the total number and percent cover of species in eight 25 cm × 25 cm quadrats along four randomly placed transects within each density subplot, and combined these 32 small-scale estimates of plant cover to represent community metrics at the density subplot level. Species richness (*S*) was the total number of unique species recorded within the density subplot. We calculated Pielou’s species evenness (*J*), where *P_i_* is the relative contribution of *i*th species to total cover, and *S* is species richness [*J* = -Σ (*P*_i_^∗^ ln(*P*_i_)/ln (*S*)], i.e., Maron et al., 2014). Abundance observations were combined for the density subplot by converting cover estimates to area approximations (e.g., 80% of a 25 cm × 25 cm quadrat = 5 cm^2^) and summing them for each species.

## Data Analysis

We used R v.3.3.1 (R Core Team, 2016) for all analyses. Inconsistent *Miscanthus* survival through time and among plots precluded an exact maintenance of original planting densities. Thus, *Miscanthus* density in analyses was represented as the average number of *Miscanthus* plants per m^2^ from 2011 to 2014 within the 4 m × 4 m density subplot. Although many plots had fewer individuals than originally planted by the end of the experiment, a significant difference in average plant density persisted among the different treatment subplots (*p* < 0.001 for mean density and pairwise comparisons, Supplement Figure 2). We incorporated replants into survival estimates as additional plants included in the summation of the total number of individuals planted per plot.

### Habitat Characteristics

We used non-metric multidimensional scaling (NMDS) to display the range of site variation in environmental variables (function *metaMDS*, *vegan* package, with Gower metric dissimilarities to account for variables measured at different scales), and its relation to plant survival per single species plot (overlaid using *ordisurf* function, see below for calculation). We evaluated the correlation strength of five environmental parameters (PARA, gravimetric soil moisture, soil redox potential, transmittance, and composite soil fertility) with the NMDS axis scores (*envfit* function), and plotted the significant habitat vectors with an *r*^2^ greater than 0.5. Because these data were analyzed at the plot level, density was not included as a factor.

### Recruitment

We quantified potential recruitment differences between habitats as: (1) overwintering seed viability (*M. sinensis and M. giganteus*); and (2) field emergence (*M. sinensis*). We analyzed mean recruitment differences between habitats separately for each species using linear mixed effects models (function *lme* in the *nlme* package) with plot nested within site as a random effect.

### Demographic Performance of *Miscanthus*

We quantified overall survival as the proportion of total individuals planted per plot that were alive in 2014. This total number of individuals planted per plot includes the initial 29 plants established in 2010, as well as any replants introduced in 2011 or 2012. Because these data were analyzed at the plot level, density was not included as a factor. We evaluated the influence of plant density on the resultant area occupied by *Miscanthus* in subplots by the end of the experiment (2014) using a linear model with pairwise Tukey tests to evaluate treatment differences.

To evaluate individual survival and growth, we analyzed differences in: (1) age-related survival probability [i.e., *g(x)* in Gotelli, 2008] and growth; and (2) plant performance between habitats. Because density had no significant effects on growth and survival parameters (see Results), it was excluded as a factor from these analyses.

#### (1) Age – Related Survival Probability and Growth

We calculated the survival probability (*p_s_*) for each plot at each successive age as:

$$p_{s}(x)=s(x)/s\left(x-1\right)$$

where *s(x)* is the cumulative survivorship at age *x*, calculated as:

$$s(x)=N(x)/N(x_{0})$$

where *N* is the number of individuals. Year 1 and 2 estimates include data from the initial planting as well as the 2011 and 2012 replantings, whereas year 3 included plants from the 2010 and 2011 cohorts. We quantified growth as the change in the maximum tiller number per plant recorded between springs each year. To avoid confounding growth estimates (which were often negative) with mortality, we only included plants that survived the full year (had non-zero tiller numbers in two successive springs, *M. giganteus*: *n* = 595; *M. sinensis*: *n* = 440).

We analyzed survival and growth at different ages with linear mixed effects models (function *lme* from R *nlme* package). Survival probability (*p_s_*) at different ages was compared at the plot level between habitat and species with plot and then year nested within site as random effects. Growth (change in tiller number) was compared at the plant level between habitat and species with random effects nested as year:plant ID:subplot:plot:site.

#### (2) Plant Performance

We intended to examine final plant tiller count, biomass in grams per surviving individual, area in m^2^, flower number and potential seed production to compare overall plant performance between habitats. However, low plant survival and lack of flowering in the floodplain forest rendered statistical habitat comparisons impossible. Therefore, we merely present the data available for differences between habitats.

### *Miscanthus* Impacts on Old Field Community Structure

Because plant survival was extremely low in floodplain forest habitats (see Results below), we limited our analysis of community impacts to the old field sites. We used a linear mixed effects model (*lme* in R *nlme* package) to examine relative impact of *Miscanthus* presence and density on community evenness and richness after 5 years (that is, in 2014). *Miscanthus* density × *Miscanthus* species were entered as fixed effects, and plot was nested within site as a random effect in the model.

We calculated the relative impact of *Miscanthus* on evenness and richness (two components of diversity) within each subplot using the relative impact (RI) equation from Vilà et al. (2006) (adapted from Armas et al., 2004).

$$\text{RI=}\frac{\left(V_{\text{control}}-V_{\text{treatment}}\right)}{\left(V_{\text{control}}+V_{\text{treatment}}\right)}$$

A negative RI value indicates an increase of the dependent variable (V, e.g., richness or evenness) associated with invader presence (positive impact of invasion). Conversely, a positive value means that invader presence decreases V (negative impact of invasion). A zero value indicates that the invader presence does not have a significant effect on the parameter (Vilà et al., 2006). The RI for each *Miscanthus* introduction subplot was computed using values from the control subplot within the same plot. To further determine whether *Miscanthus* presence significantly affected overall evenness or richness of the plant community, we performed a single sample *t*-test of the relative impact on each of these metrics within *Miscanthus* subplots with a null hypothesis of μ = 0 (i.e., RI = 0). In other words, if the mean RI value is significantly different than 0, there is a significant impact of the invader, relative to the control, for the variable measured. Further, if the RI is significant, positive values indicate greater declines in the measured variable (negative impacts) associated with invader presence. Negative RI values indicate potential increases in the variable associated with invader presence.

### *Miscanthus* Impacts on Community Composition

We examined the influence of *Miscanthus* introduction and density on community dynamics to evaluate potential implications for old field community composition and structure over time. We determined annual species turnover (shifts in community constituents) and change in species rank abundances (shifts in species hierarchies) from 2011 to 2014. Two species (*Festuca arundinacea* and *Solidago canadensis*) present in all old field plots formed the dominant matrix vegetation (>50% cover in most old field plots). We excluded these two from community dynamics analyses to maximize our ability to detect initial short term impacts on species composition.

Species annual turnover was quantified as both the proportion of species dropping out of plot censuses between 1 year and the next (‘- species’) and species added between 1 year and the next (‘+ species’) (*turnover* in R *codyn* package). Mean change in species rank was quantified as the average difference in species rank abundance between consecutive years among species that were present across the entire measurement period, and represents community shifts in relative abundance over time (Hallett et al., 2016, *rank_shift* in R package *codyn*). To account for the possibility of changes in functional group abundance over time with *Miscanthus* introduction, we examined the effect of *Miscanthus* density on the relative prevalence of four functional groups (grasses, forbs, legumes, shrub/woody species) over time (*adonis* in R *vegan* package, strata = site). We also evaluated each variable for overall impacts (RI) from *Miscanthus* presence (single sample *t*-test with null hypothesis of RI = 0).

## Results

### Habitat Characteristics

**FIGURE 1:**
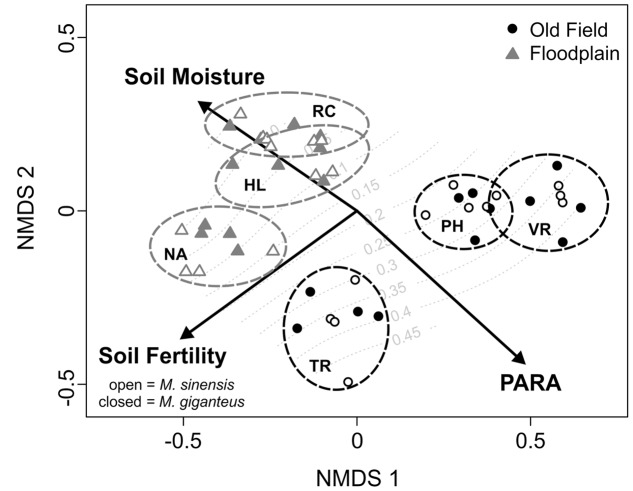
**Non-metric multidimensional scaling (NMDS) ordination of plots based on habitat variables.** Vectors indicate the direction of change in environmental factors associated with the distribution of sites in multivariate space. Final proportional survival of *Miscanthus* plantings is overlaid as a response surface in gray. Old field sites are black circles, whereas floodplain sites are gray triangles. Open points are *M. sinensis* plots, and filled points are *M. giganteus*. Sites are enclosed by dotted ovals colored according to habitat. Old fields: PH, Phillips; TR, Trelease; VR, Vermillion River. Floodplain forests: HL, Homer Lake; NA, Nanney; RC, Richter.

PARA, soil moisture, and soil fertility were strongly associated with the NMDS axis scores [*r*^2^ = 0.92 (PARA); 0.66 (soil moisture); 0.84 (soil fertility), *p* < 0.01 for all]. These variables also provide good separation between the two habitat types (**Figure 1**). Although redox potential and transmittance also significantly separated between NMDS axes, their association was weaker [*r*^2^ = 0.22 (redox); 0.32 (transmittance), *p* < 0.01 for both]. After the third year, we did not detect any *Miscanthus* in one floodplain site (Richter), and only two individuals of *M. giganteus* in another (Nanney). Several plants of both species did persist in the third floodplain site (Homer Lake). In contrast, all old field plots had many surviving individuals. Any small scale differences in how habitat characters within sites might have affected survival were obscured by the overwhelming survival difference between habitats (see below). Therefore, habitat characteristics associated with plant survival could not be statistically compared.

### *Miscanthus* Recruitment

We tested the effect of overwintering on seed germination for both *Miscanthus* species. Seed germination after overwintering differed between *Miscanthus* species; however, there was no significant effect of habitat type (*t* = -2.82; *df* = 4, 40; *p* = 0.007, **Table 1A**). Although, we could not test *M. giganteus* for habitat differences in seedling emergence, *M. sinensis* did not differ in seedling emergence between habitats (*t* = 2.12; *df* = 4, 42; *p* = 0.1, **Table 1A**).

### *Miscanthus* Demographic Performance

*Miscanthus sinensis* had both higher survival in old fields, and lower survival in floodplains, compared to *M. giganteus* (*p*_interaction_ < 0.01, df.residual = 42, *z* = -3.5, **Table 1**). *M. giganteus* survival was reduced nearly 85%, and *M. sinensis* survival over 90%, in floodplain forests compared to old fields (**Table 1B**). Overall, survival differed substantially between habitats for both species (**Figure 1**; df.residual = 20, *M. giganteus*: *p* < 0.01, *z* = -3.1; *M. sinensis*: *p* < 0.01, *z* = -3.8).

**FIGURE 2:**
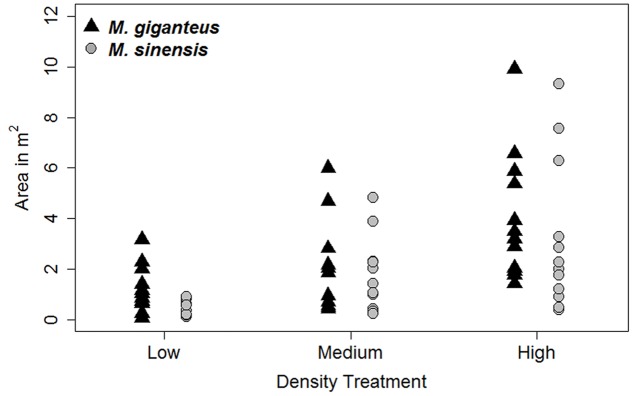
**Plot area (m^2^) occupied by *Miscanthus* species, by Density treatment, in 2014**.

**FIGURE 3:**
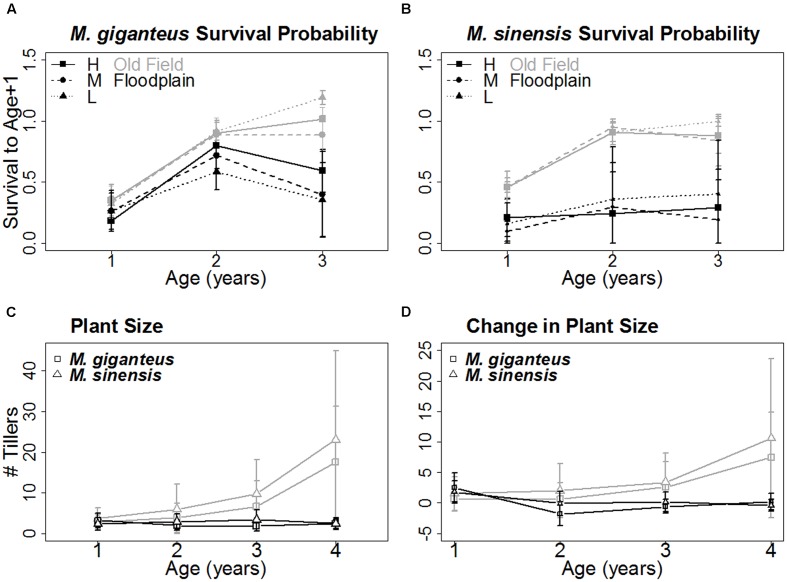
**Age-related survival and growth of each species (Mean ± SD).** The probability of surviving to the next year increased similarly with age for both *M. giganteus* **(A)** and *M. sinensis* **(B)** in old field (gray) but not floodplain (black) habitats. Size, represented by tiller number **(C)** and growth, represented by change in tiller number **(D)**, with age followed a similar pattern. Both increased with age in old field but not floodplains for both *M. giganteus* (squares) and *M. sinensis* (triangles). The *x*-axis for **(A)** and **(B)** represents the three yearly age transitions. The *x*-axis for **(C)** and **(D)** represents years of age.

Increased planting density did increase the area of the plot occupied by *Miscanthus* species by 2014 (**Figure 2**). There was a significant difference in *Miscanthus* area with density treatment (*p* < 0.001, *F* = 5.44 on 5 and 64 *df*); however, the low and medium treatments were not significantly different from each other in final *Miscanthus* area (*p* > 0.01 for high versus low and medium; *p* = 0.18 for low versus medium) by 2014. Additionally, there was no difference between *Miscanthus* species (*p* = 0.28). There was also no significant effect of plant population density on either growth or survival (e.g., **Figure 3**; *p* > 0.1 for interactions of density with habitat, survival probability, and number of tillers). Density was therefore excluded from subsequent growth and survival analyses, and we present growth and survival at the whole plot level (out of 29 individuals, plus replants, planted in each single species plot).

#### Age – Related Survival Probability and Growth

*Miscanthus* survival probability with age and habitat did not differ between species (age: *p* = 0.08, *t* = -1.8, 92 *df*; habitat: *p* = 0.24, *t* = -1.4, 4 *df*). However, age-related survival did differ between habitats (*p*_habitat^∗^age_ = 0.02, *t* = -2.4, 92 *df*; **Figures 3A,B**). Survival probability for both species increased with age in old fields, but not floodplain forests.

Growth displayed a similar pattern. Both the number of tillers and change in tiller number tended to increase with age in old fields, but not floodplain forests (*p* < 0.01, *z* = -5.1, 282 *df*; **Figures 3C,D**).

#### Final Plant Performance

Performance of surviving plants also varied significantly between habitats (**Table 1B**). Biomass and final tiller numbers were both greater in old field habitats relative to floodplain forests. However, on average, individual plant area was not. Within years, plants detected in the spring were consistently present (though sometimes with fewer tillers) in the fall. Most mortality occurred from fall to spring: for instance, there were no live plants recorded in the Richter (floodplain) site after fall 2012. Estimated reproduction did vary between species, but no plants flowered in the floodplain habitat. Therefore, old field habitats not only supported plant persistence, but also greater plant size and reproductive biomass through time compared to floodplain forests.

### *Miscanthus* Impacts on Community Structure

*Miscanthus* presence was associated with marginal increases in richness relative to control plots (*p* < 0.001, *t* = -3.90, 71 *df*, **Table 2A**). Excluding *Miscanthus* additions, introduction plots had 1.8 (±0.4 SE) species more than control plots. Relative richness impacts (RI) were unrelated to *Miscanthus* species identity (*p* = 0.46, *t* = -0.74, 46 *df*) or average density within the subplots (*p* = 0.57, *t* = -0.56, 46 *df*) compared to controls. Similarly, *Miscanthus* presence did not affect evenness (*p* = 0.98, *t* = -0.03, 71 *df*), regardless of *Miscanthus* species (*p* = 0.83, *t* = 0.21, 46 *df*, **Table 2B**) or density (*p* = 0.06, *t* = -1.9, 46 *df*). Overall, we did not detect strong impacts of *Miscanthus* introduction on community structure metrics.

**Table 2:** Richness (**A**, # of species) and Evenness (**B**, *J*) for control (no *Miscanthus*) and Treatment (*M. giganteus* or *M. sinensis* addition) plots in each of the three old field sites.

Site	(A) Richness			(B) Evenness		
		
	Control	*M. giganteus*	*M. sinensis*	Control	*M. giganteus*	*M. sinensis*
Phillips Tract	7.1 ± 1.3	7.0 ± 0.3	8.6 ± 1.0	0.49 ± 0.03	0.5 ± 0.03	0.5 ± 0.03
Trelease Prairie	14.5 ± 1.3	18.6 ± 1.7	17.3 ± 0.7	0.64 ± 0.03	0.7 ± 0.02	0.6 ± 0.03
Vermillion River	18.0 ± 1.0	18.3 ± 0.6	19.8 ± 1.1	0.66 ± 0.03	0.6 ± 0.02	0.7 ± 0.01


### *Miscanthus* Impacts on Community Composition

**FIGURE 4:**
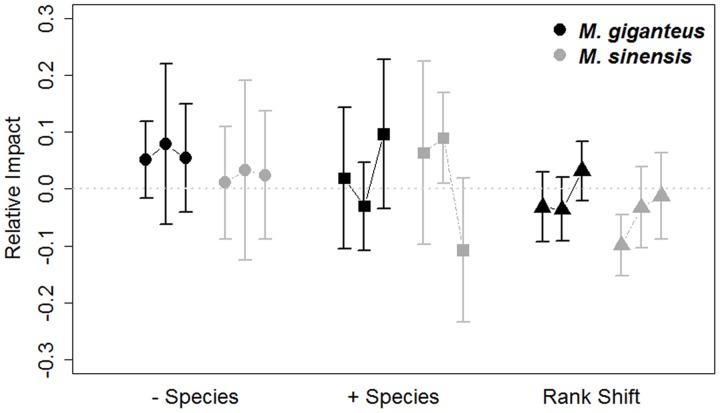
**Relative impacts (RI, Mean ± SE) on species turnover and species ranks in *M. giganteus* (black) and *M. sinensis* (gray) plots each year from 2011 to 2014.** The values for each of the three consecutive year-to-year transitions are connected by lines for each species. The gray dotted line indicates RI value of 0, indicating no impact. Positive RI values indicate greater declines in measured variables relative to gain and can be interpreted as negative associations with *Miscanthus* addition. Species turnover differed from year-to-year [+ species (appearances) = triangles, – species (disappearances) = circles], but mean shifts in species rank (squares) did not. *Miscanthus* introduction did not influence these community metrics, regardless of species.

Species turnover did vary by year (- species: *p* < 0.001, *t* = -3.8; + species: *p* = 0.002, *t* = 3.1; 189 *df*). Additionally, turnover was unaffected by *Miscanthus* species identity (- species: *p* = 0.9, *t* = -0.2; + species: *p* = 0.2, *t* = -0.1; 92 *df*) or density (- species: *p* = 0.4, *t* = 0.8; + species: *p* = 0.2, *t* = -1.2; 189 *df*). *Miscanthus* introduction in general did not appear to exclude species (RI - species: *p* = 0.12, *t* = 1.55, 215 *df*), or increase new species occurrences (RI + species: *p* = 0.4, *t* = 0.8, 215 *df*; **Figure 4**) relative to control plots.

**FIGURE 5:**
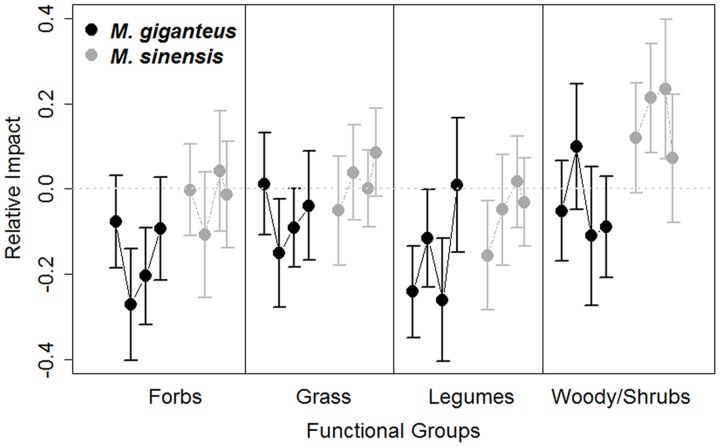
**Relative impact (RI, Mean ± SE) of *Miscanthus* introduction on the proportion abundance of functional group cover in *M. giganteus* (black) and *M. sinensis* (gray) plots each year from 2011 to 2014.** Each dot represents consecutive single year values, connected by lines for each species. The gray dotted line indicates RI value of 0, indicating no impact. Positive RI values indicate increasingly negative effects of the invader. Functional abundance did vary among years and between *Miscanthus* species, with *M. sinensis* displaying slightly stronger RI.

*Miscanthus* introduction did increase species rank shifts relative to control plots overall (RI rank shift: *p* = 0.03, *t* = -2.1, 215 *df*). However, mean species rank shift did not differ between years, species or with *Miscanthus* densities (linear model: Year: *p* = 0.6, *t* = 0.5, 189 *df*; Species: *p* = 0.2, *t* = 1.4, 92 *df*; Density: *p* = 0.3, *t* = 1.0, 189 *df*). The abundance of functional groups within plots varied by year and between *Miscanthus* species (Year: *p* = 0.006, *F* = 2.5, 1 *df*; Species: *p* = 0.004, *F* = 4.3, 1 *df*; **Figure 5**), but did not vary with *Miscanthus* density (*p* = 0.4, *F* = 4.3, 1 *df*). Overall, *Miscanthus* had some association with relative abundance of species and functional groups in the community. In addition to *Miscanthus*, introduction subplots had around two species more relative to control subplots, and RI varied by year and species. *Miscanthus* did not appear to exclude species from the community.

## Discussion

*Miscanthus* will likely escape and establish in habitats surrounding biomass production fields, but whether these introductions lead to negative community impacts remains uncertain given the limited short term empirical data available. Both floodplain and old field habitats studied were susceptible to early invasion (e.g., successful emergence and seed persistence), but differed greatly in persistence of established plants. We did observe some shifts in relative species and functional group abundances associated with *Miscanthus* presence in old field habitats. However, there is not a clear pattern indicating whether these shifts may eventually drive emergent impacts such as competitive exclusion with increasing *Miscanthus* dominance (i.e., MacDougall and Turkington, 2005; Gaertner et al., 2014).

We implemented controlled introductions of various *Miscanthus* densities in different sites and habitats to consider potential invasion success under a range of conditions. Successful establishment and increase in survivorship with age suggests *Miscanthus* can become community constituents in old field habitats. Surveys of naturalized *M. sinensis* and congener *M. sacchariflorus* (the two parent species of *M. giganteus*) in the US and Canada have associated these species with open and disturbed habitats, such as roadsides, agricultural field margins, and forest and residential property edges (Bonin et al., 2014; Dougherty et al., 2014; Hager et al., 2015b; Smith et al., 2015). Similarly, our sites were mowed annually, which likely improved conditions for establishment and growth. We found that species and functional group abundances in the resident community were affected by *Miscanthus* introductions into old fields. However, relative impacts on these community variables were not strong. This may be due in part to the site history of our old field communities. Previous invasions or disturbances can remove sensitive species and create a ‘hardier’ community that is resistant to the effects of subsequent, functionally similar invaders (i.e., Ricciardi et al., 2013). All three of our old field sites are heavily dominated by a matrix of *Festuca arundinacea* and *Solidago canadensis*, and were mowed annually, which likely improved conditions for the dominant species and establishment and growth of *Miscanthus.* This combination of dominance and disturbance could have already imposed limitations on the subdominant species assemblage present.

We noted that species richness was slightly higher when *Miscanthus* was added, and that there were some shifts in functional groups. This observation may illustrate the complexity of potential community impacts of *Miscanthus*. It is possible that *Miscanthus* can eventually reduce local dominance by key matrix species, thus opening spaces for other less frequent species. Such complex dynamics might result from allelopathic effects that *Miscanthus* can exert in some systems (Chou, 2009; Hedìnec et al., 2014; Hager et al., 2015b). More broadly, novel species introductions have been found to be both positively and negatively associated with community richness patterns, and this inconsistency has been attributed to characters such as measurement scale (i.e., Shea and Chesson, 2002; Chen et al., 2010), biotic resistance (i.e., Levine et al., 2004), resource availability and disturbance (i.e., Davis et al., 2000), and community interaction strength (i.e., MacDougall and Turkington, 2005). Whether our observed impact on species and functional group abundance represents a driving interaction (as opposed to ‘passive,’ MacDougall and Turkington, 2005) requires further study. There was a great deal of variation in the direction and strength of response over time. Although the two *Miscanthus* species were not statistically different in their relative impacts, *M. sinensis* impact values did tend to be more positive (greater negative impact) compared to *M. giganteus*, and there was a complete reversal of this pattern for species appearances (+ species) from year 3 to 4 (**Figures 4**, **5**). More replicated plots along a greater diversity or disturbance gradient might show whether these contingencies reflect real differences in community impacts related to *Miscanthus* invasions.

Density of plantings had no significant influence on any of the variables measured. We expected higher colonization pressure to increase establishment and impact strength (Ricciardi et al., 2013). Planting greater numbers of *Miscanthus* did increase the population size (number of surviving individuals), and area occupied by *Miscanthus*, in higher density subplots, but did not increase individual plant survival or the likelihood of impacts. *Miscanthus* species can form dense, monotypic stands in both the native and non-native range, and in addition to high biomass accumulation, litter accumulation is extensive (Stewart et al., 2009; Dougherty et al., 2014; Hager et al., 2015b). It may be that the higher density of individuals would have led to greater effects over time. Additionally, because early planting mortality was high and required replanting to preserve initial density treatments, plant ages varied by up to 2 years within some plots by the end of the experiment. As such, our ability to detect the impact of increased *Miscanthus* density on plant community dynamics was likely somewhat obscured by this difference in times available for biomass accumulation and, thus, plant size, within plots of similar density. It was only in the last year of the experiment that plantings, particularly of *M. giganteus*, had expanded enough spatially to grow together. Our plantings are on par with agronomic studies that suggest *Miscanthus* can take up to 4 years to reach peak biomass and canopy closure (Anderson et al., 2011; Arundale et al., 2014), though our stands were relatively diffuse compared to agronomic situations. A longer time period than our 5-year experiment would be required to fully address community consequences of *Miscanthus* introduction. Previous studies have shown that *Miscanthus* species can form dense patches outside of cultivation (e.g., Quinn et al., 2010). This lag in *Miscanthus* development provides an opportunity to eradicate escapes before their potential impacts are maximized.

Because of the potential for *Miscanthus* rhizome fragmentation and dispersal (Mann et al., 2013; West et al., 2014b), we were particularly concerned about invasive *Miscanthus* populations in floodplain forests as hidden engines of invasion. However, floodplain forests were more resistant to *Miscanthus* invasion than old field sites, inflicting greater mortality and supporting less growth over time. This difference between habitats is in contrast to previous studies in California, where *M. giganteus* established and persisted well in riparian areas and wet conditions, but declined post-establishment in drier upland sites (Barney et al., 2012; Mann et al., 2013). This discrepency is probably partially due to different ranges of moisture stress variation, as *Miscanthus* is climatically suited to broader areas of eastern compared to western North America (see Hager et al., 2014). Two of our three floodplain sites were heavily dominated by particular species before *Miscanthus* introduction (Nanney site: *Phalaris arundinacea* [reed canary grass]; Richter site: *Urtica dioica* [stinging nettle]), which likely drove microclimate variables such as light availability. This *in situ* competitive pressure may have influenced the lack of (Richter site) or very low (Nanney site) long term *Miscanthus* establishment. For instance, Hager et al. (2015a) found stronger *Miscanthus* seedling establishment limitation within forests compared to forest margins in central Illinois. However, our third floodplain site (Homer Lake) also had low *Miscanthus* persistence and no flowering over time without dominating understory species. Additionally, although *Miscanthus* performed worse in the shadier floodplain environments, previous studies have found introduced *Miscanthus* species to be relatively shade tolerant (Matlaga et al., 2012; Quinn et al., 2012). Although we lack specific site-level data on flooding frequency, spring floods in our sites resulted in high silt deposition and extended periods of standing water and saturated soils, which may have further decreased the viability of introductions. For example, 11 USGS gauging stations along waterways within an 80 km radius of our field sites recorded over-bankfull averages of 0.63–148.5 days/year between 1993 and 2012 (see Supplementary Material in West et al., 2014b). Shade and persistent flooding are likely limiting factors to *Miscanthus* establishment in this habitat, especially where apparent ground layer plant competition was low.

There are major gaps in our understanding of how species traits and characteristics of the recipient environments interact to affect community consequences from species introductions. The likelihood that an alien plant introduction will lead to community impacts depends largely on the combination of species traits and response variables measured, but whether there is an increase or decrease in the variable examined depends on environmental context (Hulme et al., 2013; Fried et al., 2014). Establishment studies alone cannot adequately identify factors that produce variation in invader impact at the community level, or their importance in predicting the impact of a plant introduction in a given community. Species invasiveness and community or habitat invasibility are often treated independently in invasion biology, and their interaction lends uncertainty to predictions about new invasions (Bennett et al., 2012; Rejmánek et al., 2013). Linking habitat context to the establishment and persistence of biomass species allow us to refine and optimize model predictions and best practices to limit invasions. Although regulations within the Renewable Fuel Standard to minimize the risk of spread from bioenergy cropping systems (US Environmental Protection Agency [EPA], 2013) do not currently apply to *Miscanthus*, large scale production of *Miscanthus* species will benefit from recommendations for proactive control. Assessing the emergent community impacts facilitates prioritization and implementation of species management. Recent models examining *Miscanthus* spread based on species characters and landscape configurations suggest spread could be rapid and extensive, and that passive management options such as buffers are insufficient to curtail dispersal (West et al., 2014b; Muthukrishnan et al., 2015; Pittman et al., 2015). Further, both *Miscanthus* species we studied can add biomass quickly and increase survivorship with age, and have a seedbank of at least 1 year (Hager et al., 2015a). Our study suggests there is a temporal window of at least 5 years in duration, and possibly longer, in which active management to monitor escapes and reduce establishment outside of cultivation (early detection and rapid response) will minimize invasion risks and community impacts associated with *Miscanthus* cultivation in the landscape. Active management, applied to passive measures such as buffers, might provide adequate control of *Miscanthus* invasion, but such combined strategies have not been modeled, to our knowledge.

The time required for escapes to accumulate in the landscape depends on the degree of active management. Thus, management cost and efficiency can indirectly drive time to escape, and the length of the escape time lag influences the relative costs and benefits of introduction (Yokomizo et al., 2012). However, lack of consistent long-term evidence of *in situ* invasiveness makes it difficult to distinguish whether a species is unlikely to be invasive, or is a future invader building toward future problems (Davis et al., 2010; Flory et al., 2012; Larkin, 2012). Field studies such as ours that evaluate establishment likelihood and consequences are vital to informing long term risk assessment and costs associated with introduction decisions. *Miscanthus* species can form nearly monotypic stands in North America, and current climate matching (Miguez et al., 2012; Hager et al., 2014; Evans et al., 2015) and landscape models (Muthukrishnan et al., 2015; Pittman et al., 2015) suggest there are few barriers to their further spread. Managing *Miscanthus* escapes in the short-term by containing or eradicating escapes will reduce the likelihood of local impacts that expand to larger- scale effects over time, which would improve the long-term benefits of perennial *Miscanthus* crops.

## Author Contributions

All authors contributed to the development, design, and initial analysis of the experiment. AD, DM, GS, NW, and RM curated the experiment and data collection. NW analyzed the data. AD, DM, GS, JF, NJ, NW, and RM wrote the manuscript.

## Conflict of Interest Statement

The authors declare that the research was conducted in the absence of any commercial or financial relationships that could be construed as a potential conflict of interest. The reviewer CH and handling Editor declared their shared affiliation, and the handling Editor states that the process nevertheless met the standards of a fair and objective review.
