# Supplementary material for: Lack of Impacts during Early Establishment Highlights a Short-Term Management Window for Minimizing Invasions from Perennial Biomass Crops
Source: Front Plant Sci. 2017 May 15;8:767. doi: 10.3389/fpls.2017.00767 (PMC5430074; doi:10.3389/fpls.2017.00767)

Supplement Figure 1. Example of the experimental setup. (A) Schematic of experimental plots within a single site. Gray versus white plots indicate the two different *Miscanthus* species. Letters indicate (L)ow, (M)edium, (H)igh density *Miscanthus* plantings and (C)ontrol subplots within each plot (B). The crosshatched square in (b) represents the seedling cage, and the circle outside the plot marks the overwintering seed plots.

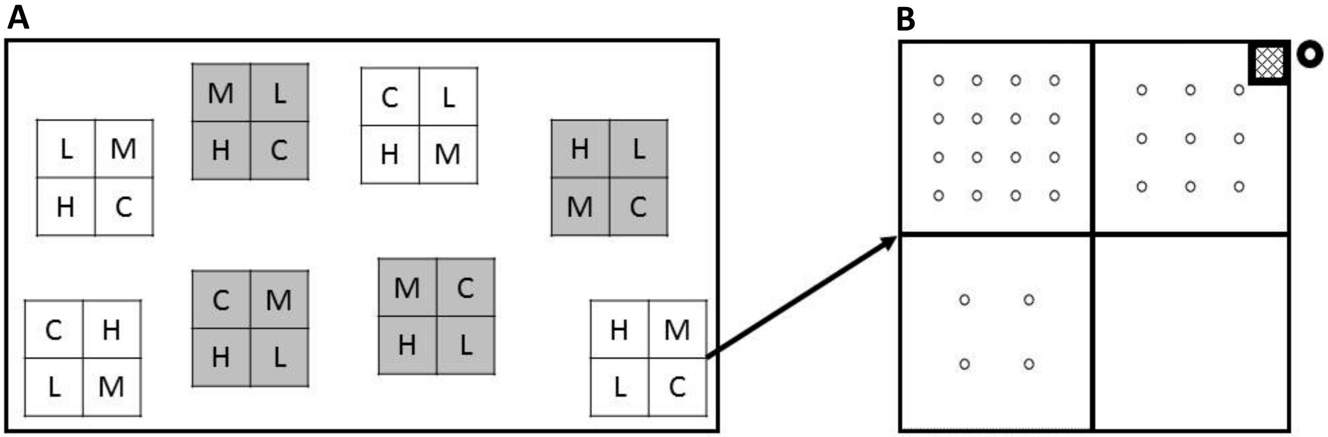

Supplement Figure 2. Average density (number of *Miscanthus* individuals within each 4x4 –m density subplot) within *Miscanthus* density treatments over the time period of the introduction experiment. Inconsistent *Miscanthus* survival through time and among plots precluded an exact maintenance of original planting densities (Low: n = 4; Medium: n = 9; High: n=16). Thus, in analyses, *Miscanthus* density was incorporated as the average number of *Miscanthus* plants per m^2 between 2011-2014 within the 4 × 4 m density subplot. Although many plots had fewer individuals than originally planted by the end of the experiment, a significant difference in average plant density persisted among the different treatment subplots ($p<0.001$, $F = 25.3$ on 5 and 64 df for a linear model evaluating average density and pairwise comparisons).There was also no difference between *Miscanthus* species in average density ($p = 0.46$).

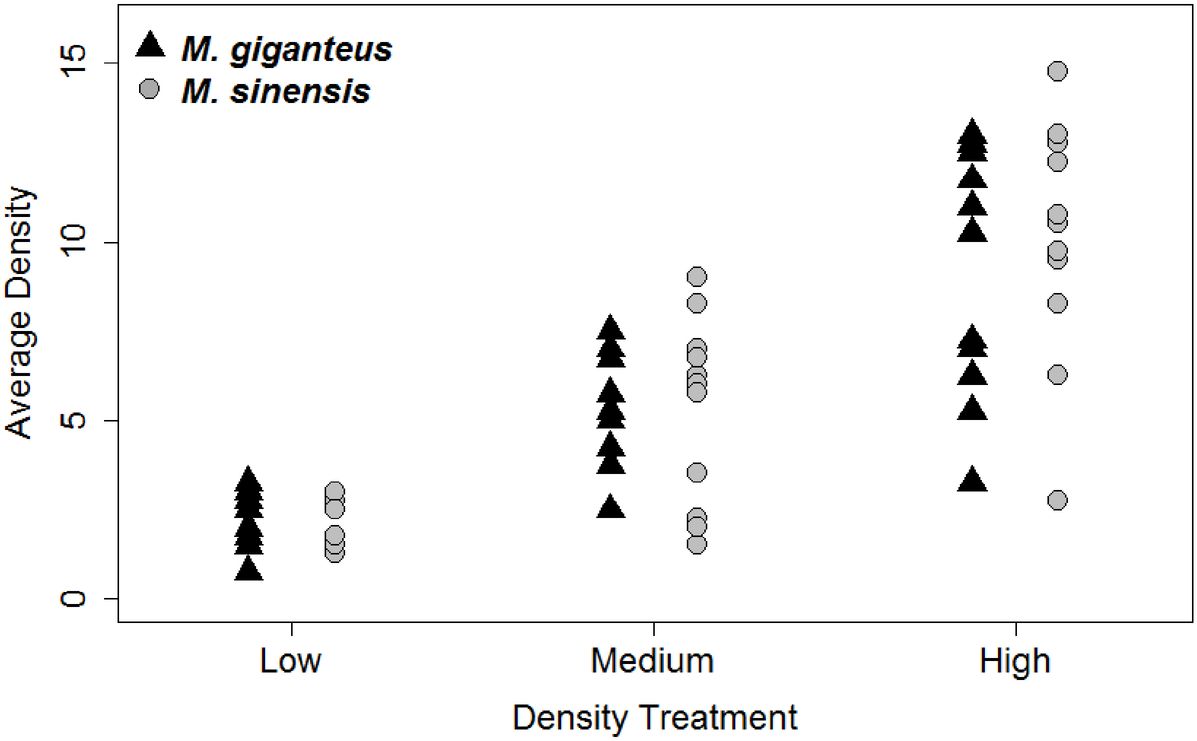

Supplement: Supplementary file 1 [file Presentation_1.PDF]
